# Supplementary material for: Bayesian refinement of protein structures and ensembles against SAXS data using molecular dynamics
Source: PLoS Comput Biol. 2017 Oct 18;13(10):e1005800. doi: 10.1371/journal.pcbi.1005800 (PMC5662244; doi:10.1371/journal.pcbi.1005800)
Supplement: S1 Archive — (BZ2) [file pcbi.1005800.s011.tar.bz2 › gromacs_4.62_bayesian_saxs_refi/share/html/online/g_rotmat.html]

g\_rotmat


| g\_rotmat Main Table of Contents |

**VERSION 4.6  
Sat 19 Jan 2013**

---

### Description

g\_rotmat plots the rotation matrix required for least squares fitting
a conformation onto the reference conformation provided with
-s. Translation is removed before fitting.
The output are the three vectors that give the new directions
of the x, y and z directions of the reference conformation,
for example: (zx,zy,zz) is the orientation of the reference
z-axis in the trajectory frame.

This tool is useful for, for instance,
determining the orientation of a molecule
at an interface, possibly on a trajectory produced with
trjconv -fit rotxy+transxy to remove the rotation
in the x-y plane.

Option -ref determines a reference structure for fitting,
instead of using the structure from -s. The structure with
the lowest sum of RMSD's to all other structures is used.
Since the computational cost of this procedure grows with
the square of the number of frames, the -skip option
can be useful. A full fit or only a fit in the x-y plane can
be performed.

Option -fitxy fits in the x-y plane before determining
the rotation matrix.

### Files

| option | filename | type | description |
| --- | --- | --- | --- |
| **-f** | traj.xtc | Input | Trajectory: xtc trr trj gro g96 pdb cpt |
| **-s** | topol.tpr | Input | Structure+mass(db): tpr tpb tpa gro g96 pdb |
| **-n** | index.ndx | Input, Opt. | Index file |
| **-o** | rotmat.xvg | Output | xvgr/xmgr file |

### Other options

| option | type | default | description |
| --- | --- | --- | --- |
| **-[no]h** | bool | no | Print help info and quit || **-[no]version** | bool | no | Print version info and quit || **-nice** | int | 19 | Set the nicelevel || **-b** | time | 0 | First frame (ps) to read from trajectory || **-e** | time | 0 | Last frame (ps) to read from trajectory || **-dt** | time | 0 | Only use frame when t MOD dt = first time (ps) || **-[no]w** | bool | no | View output .xvg, .xpm, .eps and .pdb files || **-xvg** | enum | xmgrace | xvg plot formatting: xmgrace, xmgr or none || **-ref** | enum | none | Determine the optimal reference structure: none, xyz or xy || **-skip** | int | 1 | Use every nr-th frame for -ref || **-[no]fitxy** | bool | no | Fit the x/y rotation before determining the rotation || **-[no]mw** | bool | yes | Use mass weighted fitting |

---

http://www.gromacs.org  
gromacs@gromacs.org
